# Impact of Dietary Practices on DNA Adduct Formation by Aristolochic Acid I in Mice: Drinking Alkaline Water as a Risk Mitigation Strategy

**DOI:** 10.1021/acs.chemrestox.5c00354

**Published:** 2025-12-08

**Authors:** Hong-Ching Kwok, Jiayin Zhang, Nikola M. Pavlović, Wan Chan

**Affiliations:** a Department of Chemistry, 58207 The Hong Kong University of Science and Technology, Clear Water Bay, Kowloon, Hong Kong; b Medical Faculty, University of Niš, Bulevar Dr Zorana Đinđića 81, Niš 18000, Serbia

## Abstract

Balkan endemic nephropathy (BEN) is a chronic kidney disease associated with the consumption of aristolochic acids (AAs) through contaminated food sources. AAs are known to form DNA adducts that are implicated in tumorigenesis and kidney fibrosis. Given the sensitivity of DNA adduct formation to dietary factors, this study aimed to investigate the impact of various dietary practices on AA-DNA adduct formation, thereby assessing the risk of developing BEN. We quantified AA-DNA adducts in DNA extracted from the kidneys and livers of mice subjected to high-fat, high-protein, high-sucrose, and high-salt diets, utilizing a highly sensitive liquid chromatography–tandem mass spectrometry method combined with stable isotope dilution. Our results demonstrated that unbalanced diets significantly elevated the formation of DNA adducts from AAs. Notably, mice fed high-fat diets exhibited increases in adduct levels of 71 and 114% for diets containing 17 and 25% fat, respectively. Mice on a 20% sucrose diet showed an 80% increase in adduct levels compared to those on a standard diet. Further investigations using gut sacs from the small intestines of these mice revealed that the increased level of DNA adduct formation was primarily attributed to enhanced intestinal absorption. Additionally, we observed that drinking alkaline water reduced adduct levels by 30% compared to tap water, likely by decreasing AA absorption. In contrast, commonly used dietary supplements, such as vitamin C and cysteine, significantly increased AA-DNA adduct levels by enhancing the activity of enzymes involved in the metabolic activation of AAs. These findings highlight the critical role of a balanced diet in mitigating the risk of BEN and suggest that alkaline water consumption may serve as a protective strategy for individuals living in AA-contaminated regions.

## Graphical abstract

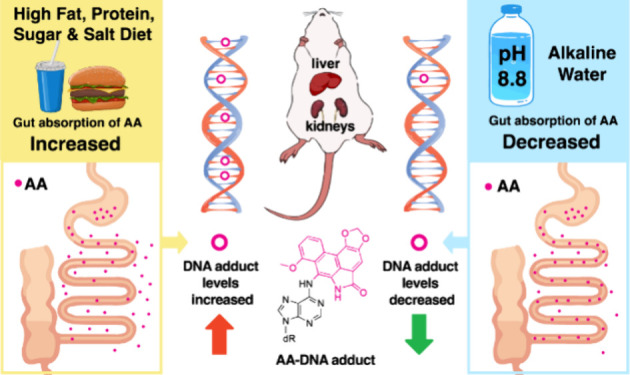

## Introduction

First identified in the 1960s, Balkan endemic nephropathy (BEN) is a multifactorial disease with a high prevalence in several countries of the Balkan Peninsula, including Bosnia and Herzegovina, Bulgaria, Croatia, Romania, and Serbia.^1–5^ Extensive research over recent decades has provided compelling evidence that chronic dietary exposure to aristolochic acids (AAs; Figure 1), derived from the widely distributed weed *Aristolochia clematitis* in the region, is the major causative agent of this disease.^1,4–9^ Key characteristics of BEN include a long incubation period and familial clustering despite the fact that it is not inherited.^1,2,7,10–12^ These features complicate the diagnosis and understanding of the disease’s transmission, underscoring the potential link between BEN and the dietary practices of affected families. This highlights the urgent need for further investigation into the poorly understood dietary practices contributing to this condition.

**Figure 1:**
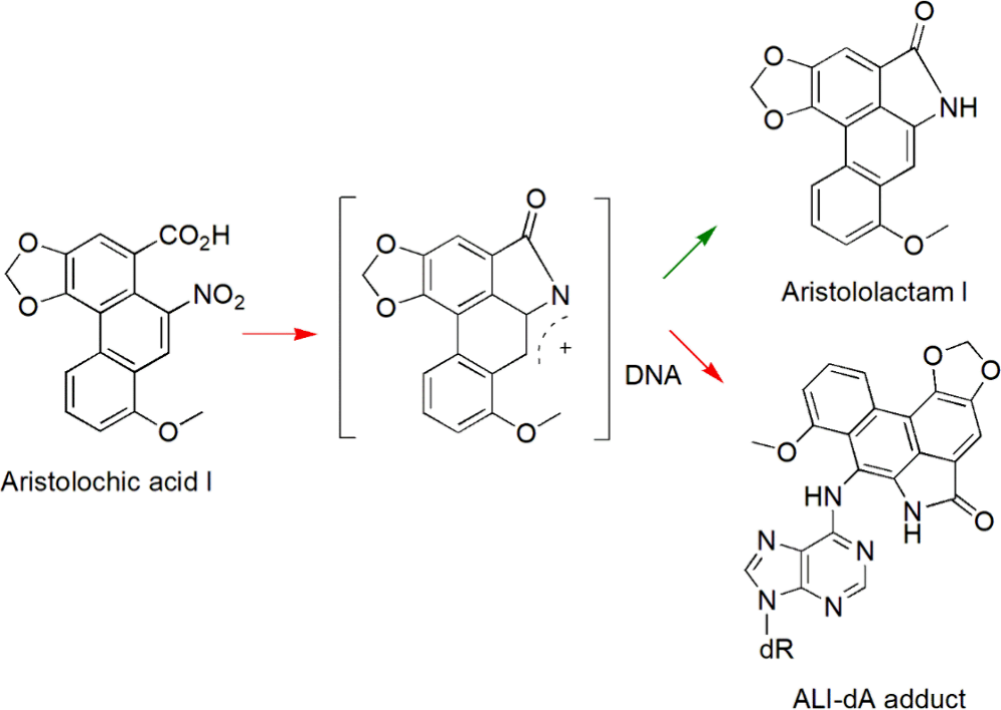
Metabolic activation and DNA adduct formation of aristolochic acid I.

Although the mechanisms have not yet been proven in laboratory animals, *in vitro* studies using cultured human cells indicate that cells exposed to elevated levels of nutrients, such as fatty acids and amino acids, produce AA-DNA adducts (Figure 1) at significantly higher frequencies than those cultured in standard cell culture medium.^13^ Notably, high levels of AT-TA transversion have been detected in both patients with BEN and laboratory animals exposed to AAs.^1,2,7,10,11,14,15^ Given that reductive metabolic activation and associated DNA damage are known to be responsible for both AA-induced tumor development and renal fibrosis,^16–18^ these findings suggest a potential causative role of dietary habits in the development of BEN. Understanding how dietary habits influence the toxicity of AAs is crucial for developing effective prevention strategies.

Building on these findings, we conducted an *in vivo* study to investigate the effects of various dietary practices on the toxicity of AAs in mice. One key reason for extending our research from cell cultures to experimental animals is that *in vitro* studies do not adequately account for differences in AA absorption in the intestines and metabolism in the liver under various nutrient conditions, which are important factors in toxicology.^19–21^ Our recent findings demonstrate that the absorptivity of AAs is a critical determinant of their toxicity,^22^ making an *in vivo* study essential for addressing this information gap.

Using liquid chromatography–tandem mass spectrometry (LC–MS/MS) coupled with a stable isotope-dilution method, we measured the levels of 7-(deoxyadenosin-N^6^-yl)-aristolactam I (ALI-dA), which is the most abundant and mutagenic AA-DNA adduct,^7,10^ in both target and nontarget organs of mice exposed to aristolochic acid I (AA-I) and fed high-protein, high-fat, high-salt, or high-sugar diets. Additionally, we assessed the levels of AAs and their major reductive metabolites, known as aristolactam I (AL-I; Figure 1),^22^ in blood, kidney, and liver samples. Our results indicate for the first time that a high-fat and high-sugar diet significantly enhances the formation of AA-DNA adducts, with adduct levels nearly doubling compared to those observed in mice on a standard diet.

Subsequently, we performed a noneverted gut sac experiment using isolated segments of the small intestine to assess potential differences in AA absorption among mice subjected to various dietary regimens. We observed higher AA absorption efficiency in gut sacs, especially those prepared from mice fed high-fat and high-sugar diets. Together with the increased AA levels in serum, these results suggest that the rise in adduct formation is attributed to enhanced intestinal absorption of AAs in the context of an unbalanced diet.

Surprisingly, AA-exposed mice fed commonly used health supplements such as vitamin C and cysteine exhibited increased adduct formation, likely due to the enhanced activity of NAD­(P)­H:quinone oxidoreductase 1 (NQO1), a key enzyme involved in the metabolic activation of AAs.^23–26^ Conversely, drinking alkaline water reduced adduct formation in AA-exposed mice by 30% compared to those drinking tap water, while drinking acidified water increased adduct formation by around 30%. Subsequent studies demonstrated that drinking water altered the pH of the small intestine, affecting AA absorptivity and, ultimately, the toxicity of AAs.

Overall, the results of this study highlight dietary practices as a previously unrecognized causative factor in the development of BEN and suggest that drinking alkaline water may serve as a risk mitigation strategy. Future risk mitigation efforts should prioritize changes in dietary habits to effectively reduce the disease risk.

## Experimental Section

Caution: AA-I is nephrotoxic and carcinogenic and should be handled with caution.

### Chemicals and Materials

Chemicals and reagents used were of the highest purity available and were used without further purification unless otherwise specified. AA-I was obtained from Acros (Morris Plains, NJ). AL-I, ALI-dA, 7-(deoxyguanosin-N^2^-yl)-aristolactam I (ALI-dG), and the [^15^N_5_]-labeled internal standard (^15^N_5_-ALI-dA) were from a previous study.^27–29^ Benz­[*cd*]­indol-2­(1*H*)-one, alkaline phosphatase, DNase I, and nuclease P1 were obtained from Sigma (St. Louis, MO). Venom phosphodiesterase was obtained from US Biological (Swampscott, MA). Mice chow with different fat, protein, sugar, and salt levels were purchased from Shuyushengwu, China (Table S1). LC–MS-grade methanol and acetonitrile were acquired from Tedia (Fairfield, OH). Commercial alkaline water was purchased from a local supermarket. Acidified and alkaline water was prepared by adding hydrochloric acid and sodium hydroxide, respectively, to tap water as reported previously.^30–32^ Deionized water was further purified by using a laboratory water purification system (Cascada, PALL; Port Washington, NY) and used in all experiments.

### Instrumental Analysis

The analysis of AA-I and AL-I was conducted using a 4000 QTRAP LC–MS/MS system (Foster City, CA), while the analysis of the ALI-dA adduct was performed on a Waters Xevo TQ-XS LC–MS/MS system.^13,22,27–29^ Both systems utilized positive electrospray ionization and operated in multiple reaction monitoring (MRM) mode for the analyses. A Luna C18 column (100 × 2 mm, 3 μm; Phenomenex; Torrance, CA) was used for all analyses, with the specific mobile phase composition, liquid chromatography (LC) gradient, and mass spectrometry (MS) parameters detailed in Table S2.

### Mouse Experiments

The animal protocol for this study was approved by the Animal Ethics Committee of HKUST (AEP-2023-0041) and adhered to the Animal Ordinance established by the Hong Kong Department of Health. Male C57BL/6J mice were obtained from the HKUST Laboratory Animal Facility. The mice were housed in a temperature- and humidity-controlled environment with artificial dark/light cycles, and food and water were provided ad libitum throughout the study.

#### Nutrients

After a 3 day acclimatization period, the mice (*n* = 50; 2–3 weeks old) were randomly divided into 10 groups and fed diets with varying levels of protein, fat, sugar, and salt (Table S1). Twelve weeks later, the mice were administered a single oral dose of AA-I at 10 mg/kg in a 0.1 M NaHCO_3_ solution. One group of mice (*n* = 5) fed a standard diet and administered an equal volume of the dosing vehicle was used as control. Twenty-four hours after the initiation of AA-I dosing, the mice were sacrificed by decapitation, and blood sera, kidneys, and livers were collected for analysis.

#### Drinking Water

Similarly, mice (*n* = 20; 8–9 weeks old) on a standard diet were given tap (pH 6.5), alkaline (pH 8.8), acidified (pH 3.0), and commercial alkaline (pH 8.8) water ad libitum for 2 weeks before being administered a single oral dose of AA-I at 10 mg/kg in a 0.1 M NaHCO_3_ solution. Twenty-four hours after the AA-I dosing, the mice were sacrificed, and kidneys, livers, and sera were collected for ALI-dA, ALI-dG, AA-I, and AL-I analysis, as described above.

#### Health Supplements

Alternatively, mice (*n* = 30; 8–9 weeks old) on a standard diet were administered a single oral dose of glutathione (GSH), *N*-acetyl cysteine (NAC), ascorbic acid, calcium carbonate (CaCO_3_), and cysteine for 2 weeks before being given a single oral dose of AA-I at 10 mg/kg in a 0.1 M NaHCO_3_ solution. Twenty-four hours after the AA-I dosing, the mice were sacrificed, and kidneys, livers, and sera were collected for ALI-dA, ALI-dG, AA-I, and AL-I analysis, as described above.

### Serum and Organ Preparation for AA-I and AL-I Analysis

Fifty microliters of serum was mixed with 200 μL of ice-cold acetone, vortexed, and centrifuged at 13,800 rcf at 4 °C for 10 min to precipitate blood proteins. The supernatant (180 μL) was then extracted and combined with 20 μL of a 600 nM solution of the internal standard benz­[*cd*]­indol-2­(1*H*)-one. The mixture was dried under a nitrogen stream, and the residues were redissolved in 50 μL of methanol for subsequent LC–MS/MS analysis of AA-I and AL-I.^33,34^

To determine the distribution of AA-I and AL-I in the kidney and liver, 50 mg of each organ was accurately weighed and rinsed with ice-cold PBS before homogenizing in 0.5 mL of PBS. Two hundred microliters of the homogenate was collected and processed similarly to the serum analysis. In brief, the homogenate was mixed with four times ice-cold acetone for protein precipitation, and the protein content in the sample was quantified using a Merck BCA protein assay kit (St. Louis, MO) according to the manufacturer’s protocol. The supernatants were then dried under a nitrogen stream, and the residues were resuspended in 50 μL of methanol for LC–MS/MS analysis.^27,34^

### DNA Isolation and Digestion

DNA was isolated from the kidneys and livers of AA-exposed mice using the Omega Biotek DNA isolation kit (Norcross, GA) following the manufacturer’s protocol. The isolated DNA (approximately 15 μg dissolved in 100 μL of water) was mixed with 15 μL of an internal standard solution containing 0.1 nM ^15^N_5_-ALI-dA before undergoing enzymatic digestion with nuclease P1, DNase I, alkaline phosphatase, and snake venom phosphodiesterase, as previously described.^13,22,27,28,34^ The resulting DNA hydrolysates were centrifuged at 13,800 rcf at 4 °C for 10 min before being analyzed using our previously developed LC–MS/MS method.^13,22,27,28,34^

### Noneverted Gut Sac Experiment

The noneverted gut sac experiment was conducted essentially as described previously.^22,35,36^ In brief, segments of the jejunum (5 to 8 cm) were freshly prepared from mice that had been fed diets containing different nutrients for 12 weeks (Table S1; *n* = 3) but with no AA-I treatment. The intestinal segments were rinsed with cold Tyrode’s solution and filled with 150 μL of AA-I dissolved in oxygenated Tyrode’s solution (1.0 μM; *n* = 3). The gut sacs were then placed in glass test tubes containing 8 mL of oxygenated Tyrode’s solution and maintained at 37 °C in a water bath. Samples (200 μL) of the solution outside the sacs were collected at 15, 30, 45, 60, 90, and 120 min. The samples were extracted three times with 600 μL of ethyl acetate. The extracts were dried under a nitrogen stream, and the residues were resuspended in 50 μL of 70% methanol containing 60 nM internal standard benz­[*cd*]­indol-2­(1*H*)-one. The samples were subsequently analyzed using LC–MS/MS for the determination of AAs.^22^ Similarly, gut sacs for investigating the effect of pH on AA-I absorption were prepared by filling segments of the jejunum obtained from mice on a standard diet without AA-I treatment with 150 μL of AA-I (1.0 μM; *n* = 3) dissolved in oxygenated Tyrode’s solution buffered to different pH levels (5.5, 6.0, 6.5, and 7.0).

### Statistical Analysis

All data were analyzed using the GraphPad software and are presented as the mean ± standard deviation (SD) from five or three independent experiments. Statistical comparisons between the control and experimental groups were performed using Student’s *t* test with a 95% confidence interval. Significance levels were defined as follows: ns: *p >* 0.05, *p < 0.05, **p < 0.01, ***p < 0.001, ****p < 0.0001.

## Results and Discussion

### ALI-dA Adduct Levels in Mice Fed Different Diets

While no ALI-dA adduct was detected in DNA isolated from nonexposed mice, in kidney DNA isolated from AA-I-exposed mice on a standard diet, ALI-dA was detected at a level of 68.5 ± 17 adducts per 10^6^ nucleotides. Notably, analysis of kidney DNA from mice receiving diets with elevated fat, protein, and sucrose contents revealed a significant increase in DNA adduct formation (Figure 2), indicating that an unbalanced diet enhances the nephrotoxicity and carcinogenicity of AAs.

**Figure 2:**
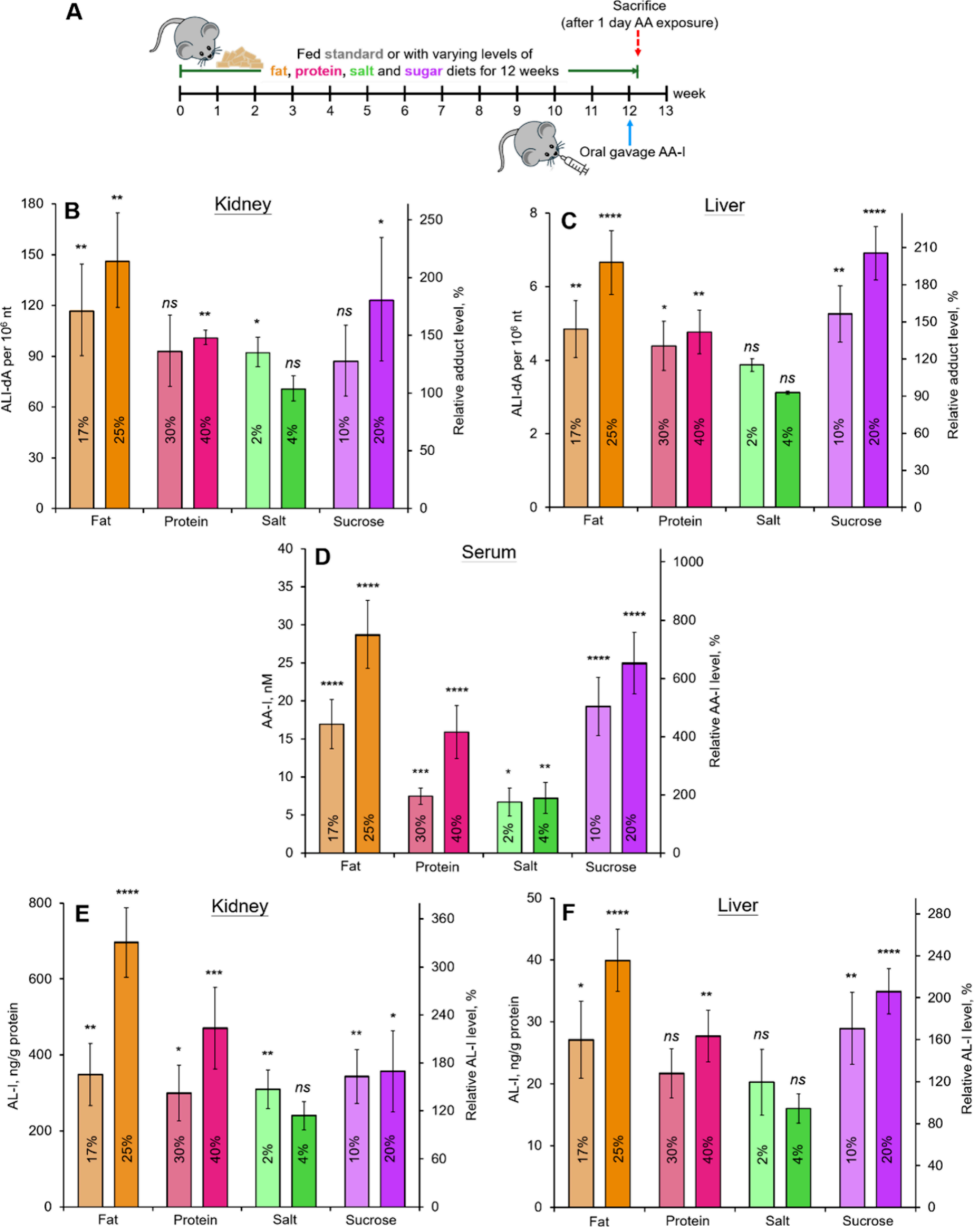
Influence of dietary practices to the DNA adduct formation in aristolochic acid I exposed mice. (A) Experimental design for aristolochic acid I exposed mice fed different nutrient-enriched diets. DNA adduct levels in the (B) kidney and (C) liver, (D) serum AA-I levels, and concentrations of aristolactam I in the (E) kidney and (F) liver of aristolochic acid I exposed mice on different nutrient-enriched diets. Data represent mean ± SD of five independent measurements and were compared with those in mice receiving the standard diet (B: 68.5 ± 17 adducts per 10^6^ nucleotides; C: 3.37 ± 0.47 adducts per 10^6^ nucleotides; D: 3.84 ± 0.69 nM; E: 210.2 ± 18.7 ng aristolactam I/g protein; and F: 17.0 ± 4.1 ng aristolactam I/g protein).

Specifically, the analysis showed a general trend of increasing ALI-dA adduct levels in the kidney DNA of mice fed nutrient-enriched diets (Figure 2B). The most significant effects were observed in mice on a high-fat diet, where adduct levels rose by 71% (117.3 ± 27.0 adducts per 10^6^ nucleotides) and 114% (146.7 ± 27.9 adducts per 10^6^ nucleotides) for diets containing 17 and 25% fat, respectively, compared to the control group on a standard diet. In contrast, kidney DNA from mice consuming diets with 40% protein and 20% sucrose exhibited increases in adduct levels of 48 and 80%, respectively. As discussed in the following section, the observed increase in DNA adduct formation from AA-I is likely due to the enhanced intestinal permeability and subsequent absorption of AA-I associated with the intake of unbalanced diets. To the best of our knowledge, this study is the first to demonstrate that an unbalanced diet can increase the toxicity of AAs.

Discrepancy was observed in mice fed a diet containing 4% sodium chloride, 30% protein, and 10% sucrose, which may be attributed to the relatively small increase in adduct levels combined with significant interindividual variation among the exposed mice.

Although detected at frequencies approximately 20 times lower than those in kidney DNA, a similar trend of increasing adduct formation was observed in liver DNA isolated from AA-I-treated mice fed nutrient-enriched diets (Figure 2C). This finding is consistent with previous observations regarding the liver being a nontarget organ for AAs.^22,27,37–39^ Nevertheless, these results shed light on some common factors on different dietary habits on AA toxicity.

In the same LC–MS/MS method, the levels of ALI-dG, another adduct formed by AA-I,^7,10^ were quantified simultaneously with ALI-dA. The analysis detected ALI-dG at concentrations at least 10 times lower than those of ALI-dA in all the digested kidney samples, while it was nondetectable in most of the liver samples. These data indicate that the different dietary conditions did not significantly affect the formation of DNA adducts of AA-I with dA and dG.

### AA-I and AL-I Levels in Serum and Organs of Mice Fed Different Diets

The similar DNA adduct patterns observed in both kidney and liver tissues of AA-I-exposed mice on different diets suggest common factors influencing AA toxicity, with the intestinal absorptivity of AA being one of the most likely causes. To test this hypothesis, we first quantified the levels of AA-I and AL-I (Figure 1), a key metabolite involved in the reductive activation of AA that forms the promutagenic ALI-dA adduct,^40–44^ in the serum of mice consuming various diets.

The analysis revealed an intriguing phenomenon: serum AA-I levels exhibited a pattern analogous to that of the ALI-dA adducts found in kidney and liver DNA (Figure 2D). These results indicate for the first time that different dietary practices have a pronounced effect on AA toxicity, i.e., by affecting the intestinal absorption of AAs. Specifically, the varying diets influenced intestinal absorption, thereby affecting the toxicity of AA to different extents, with the most significant effects observed in mice fed a high-fat diet. This finding is supported by previous studies indicating that a high-fat diet induces intestinal hyperpermeability by increasing bile acid secretion.^45^

Interestingly, AL-I was not detected in the serum samples. This absence may be attributed to its rapid elimination or phase II metabolism, resulting in the formation of conjugated metabolites shortly after it is produced in the liver.^33,46,47^ Consequently, low levels of AL-I remain for detection.

In contrast, AL-I—unlike AA-I—was detected in both kidney and liver tissues of mice fed different diets (Figures 2E,F), exhibiting a pattern similar to that of AA-I in serum (Figure 2D) and ALI-dA in kidney and liver DNA (Figure 2B,C). Probably, AL-I, because of its higher lipophilicity, is better accumulated in the organs than AA-I.^48^ These findings further emphasize the role of reductive metabolism, along with the above-mentioned intestinal absorption, in contributing to the observed differences in DNA adduct formation and thus the nephrotoxicity and carcinogenicity of AA-I influenced by dietary practices. Notably, AL-I levels in the liver were approximately 20 times lower than those in the kidney, which align excellently with the observations from the DNA adduct analysis.

### AA-I Absorption through a Noneverted Intestinal Sac Model

The AA-I levels detected in the serum of mice represent the net result of intestinal absorption, elimination, and hepatic metabolism.^49,50^ To confirm the significant role of intestinal absorption in the observed differential toxicity of AA-I in mice on different diets, we conducted an independent study using a noneverted intestinal sac model. Thus, this model allowed us to isolate the effect of intestinal absorption.^50–53^

Analysis of the receiver solution outside the sacs, collected at various time points after the experiment commenced, revealed a higher absorption rate and efficiency of AA-I from the intestines of mice on high-fat, high-protein, and high-sucrose diets compared to those on a standard diet (Figure 3A), with the highest absorption rate observed in mice on a high-fat diet. These results indicate that AA-I was absorbed more effectively from the intestinal sacs of mice consuming diets rich in fat, protein, and sucrose into the surrounding Tyrode’s solution, which aligns excellently with the observed DNA adduct and serum AA-I patterns, highlighting an important role of increased intestinal absorption of AA-I in the observed increased toxicity of AA-I.

**Figure 3:**
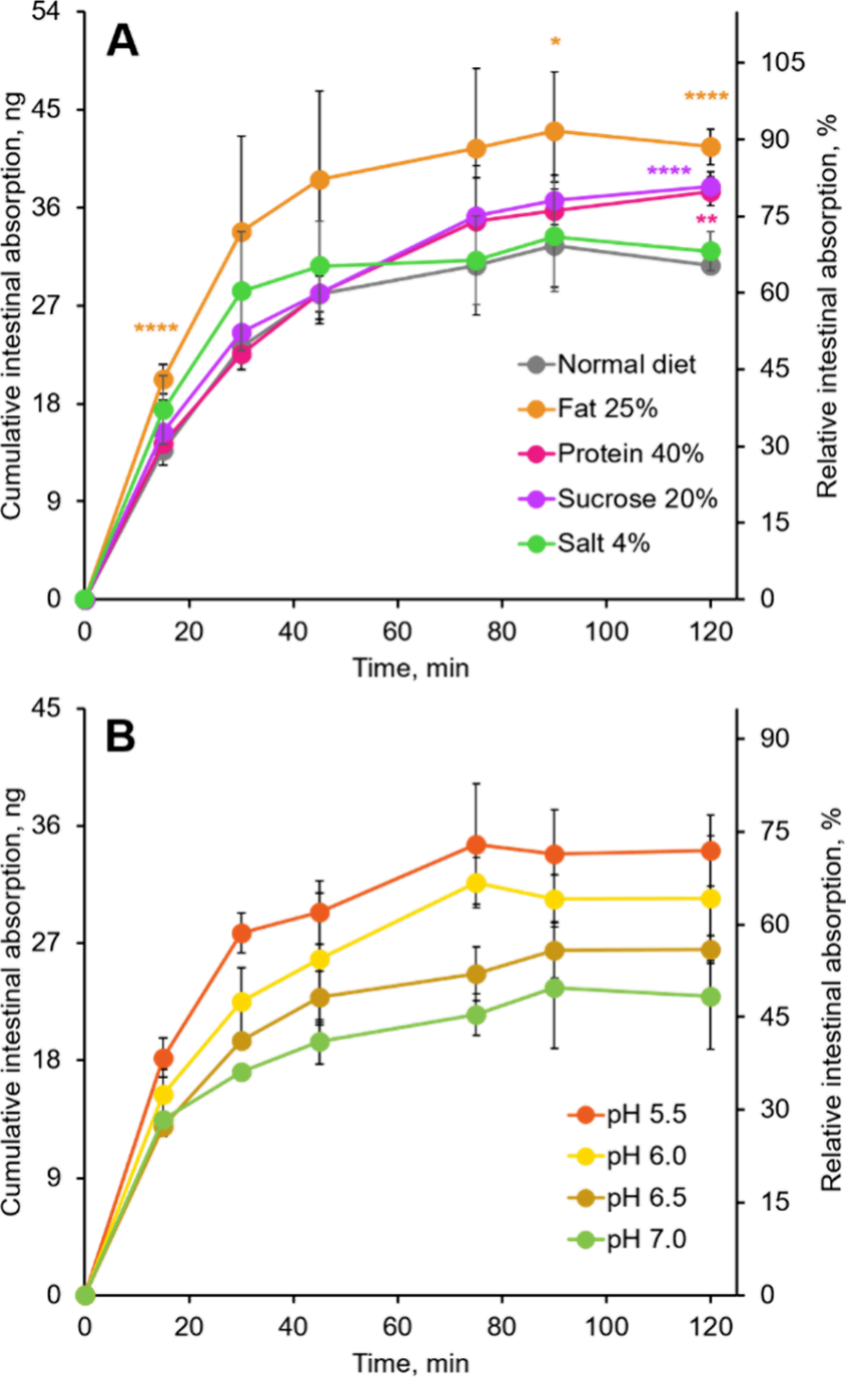
Intestinal absorption of AA-I in gut sacs prepared from mice fed diets with varying nutrient levels and at different pH levels. Absorption of AA-I into the Tyrode’s solution outside the gut sacs prepared from mice on (A) different nutrient-enriched diets and (B) the standard diet with different pH levels of Tyrode’s solution used, which contained 1.0 μM of AA-I. Statistical analyses in panel A were performed using Student’s *t* test to compare the results with those from mice receiving the standard diet, with significance levels indicated as follows: * *p* < 0.05, ** *p* < 0.01, **** *p* < 0.0001. The data represent means ± SD for three independent experiments.

### Effects of Health Supplements on the DNA Adduct Formation of AA-I in Mice

Previous *in vitro* studies have also demonstrated that certain common health supplements may be involved in the metabolic deactivation of AAs, potentially reducing their toxicity by lowering DNA adduct formation.^13^ In this study, we quantified the formation of ALI-dA adducts in mice exposed to AA-I while receiving health supplements. Specifically, we assessed DNA adduct levels in the kidneys and livers of mice on a standard diet that were orally administered NAC, GSH, cysteine, CaCO_3_, and ascorbic acid daily for 2 weeks prior to AA-I administration.

Surprisingly, analysis of kidney DNA isolated from mice fed ascorbic acid and cysteine revealed significant increases in ALI-dA adduct levels, with increases of approximately 30 and 40%, respectively (Figure 4B). In contrast, no significant differences were observed in mice fed NAC, GSH, and CaCO_3_ compared to control mice that did not receive health supplements. A similar pattern of ALI-dA adduct formation was also observed in liver DNA from the same group of mice (Figure 4C).

**Figure 4:**
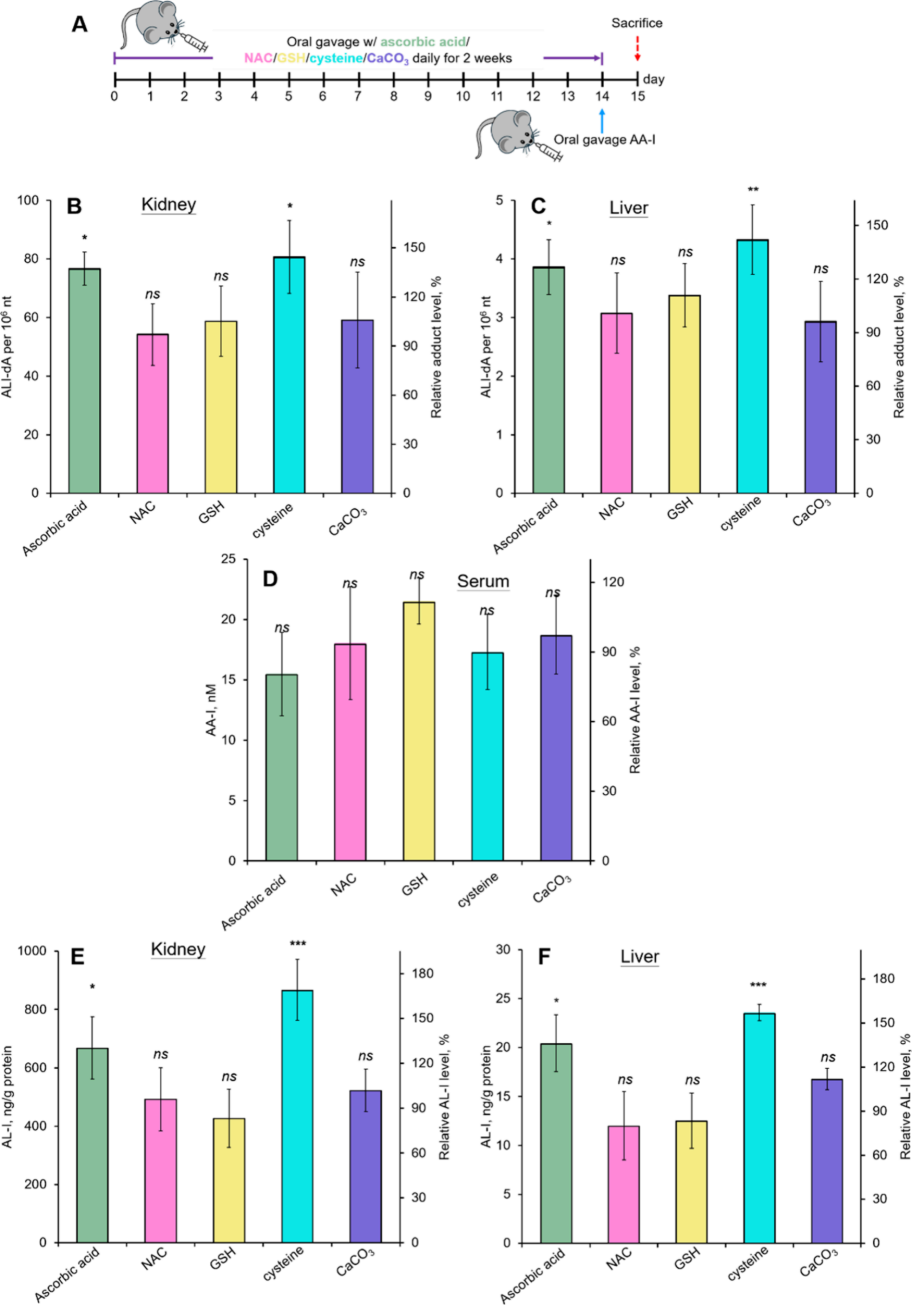
Effect of various health supplements to the DNA adduct formation in aristolochic acid I exposed mice. (A) Experimental design for aristolochic acid I exposed mice fed the standard diet with different health supplements. DNA adduct levels in the (B) kidney and (C) liver, (D) serum AA-I levels, and concentrations of aristolactam I in the (E) kidney and (F) liver of aristolochic acid I exposed mice treated with different health supplements. Date represent mean ± SD of five independent measurements and were compared with those in mice receiving the dosing vehicle (B: 56.0 ± 14 adducts per 10^6^ nucleotides; C: 3.06 ± 0.50 adducts per 10^6^ nucleotides; D: 19.3 ± 3.4 nM; E: 513.8 ± 95.4 ng aristolactam I/g protein; and F: 15.1 ± 2.9 ng aristolactam I/g protein).

While analysis of AA-I in the serum of AA-exposed mice showed no significant differences among the various health supplements compared to the control (Figure 4D), indicating that these supplements did not significantly enhance the intestinal absorption of AA-I, the analysis of AL-I in both kidney and liver tissues demonstrated a similar pattern of AL-I adduct formation as observed with ALI-dA adducts (Figure 4E,F). These results suggest that ascorbic acid and cysteine may enhance the DNA adduct formation of AA-I by increasing its metabolic activation. This observation aligns with previous studies reporting that ascorbic acid and cysteine increase the activity of NQO1, a key enzyme involved in the metabolic activation of AAs.^23–26^ Notably, it has been shown that ascorbic acid and cysteine enhance the expression of NQO1 at the transcriptional level via the Nrf2-ARE signaling pathway.^23,24^

Conversely, previous studies have indicated that ascorbic acid alleviates oxidative stress induced by AA exposure in treated mice, thereby lowering the associated cancer risk.^54^ Therefore, further research is necessary before ascorbic acid is widely recommended for mitigating the cancer risks associated with AA exposure.

### Effects of pH of Drinking Water on the DNA Adduct Formation of AA- I in Mice

An attempt was also made to investigate the effect of drinking water pH on the DNA adduct formation of AA-I in mice maintained on a standard diet. The results revealed a notable pH dependence in the formation of DNA adducts (Figure 5). Specifically, an inverse relationship between pH and ALI-dA levels was observed, with adduct levels in mice drinking alkaline water (pH 8.8) being nearly 70% of those drinking tap water (pH 6.5; Figure 5B,C). In contrast, an increase in adduct levels—130% relative to those of mice drinking tap water—was noted in mice consuming acidified water at pH 3.0.

**Figure 5:**
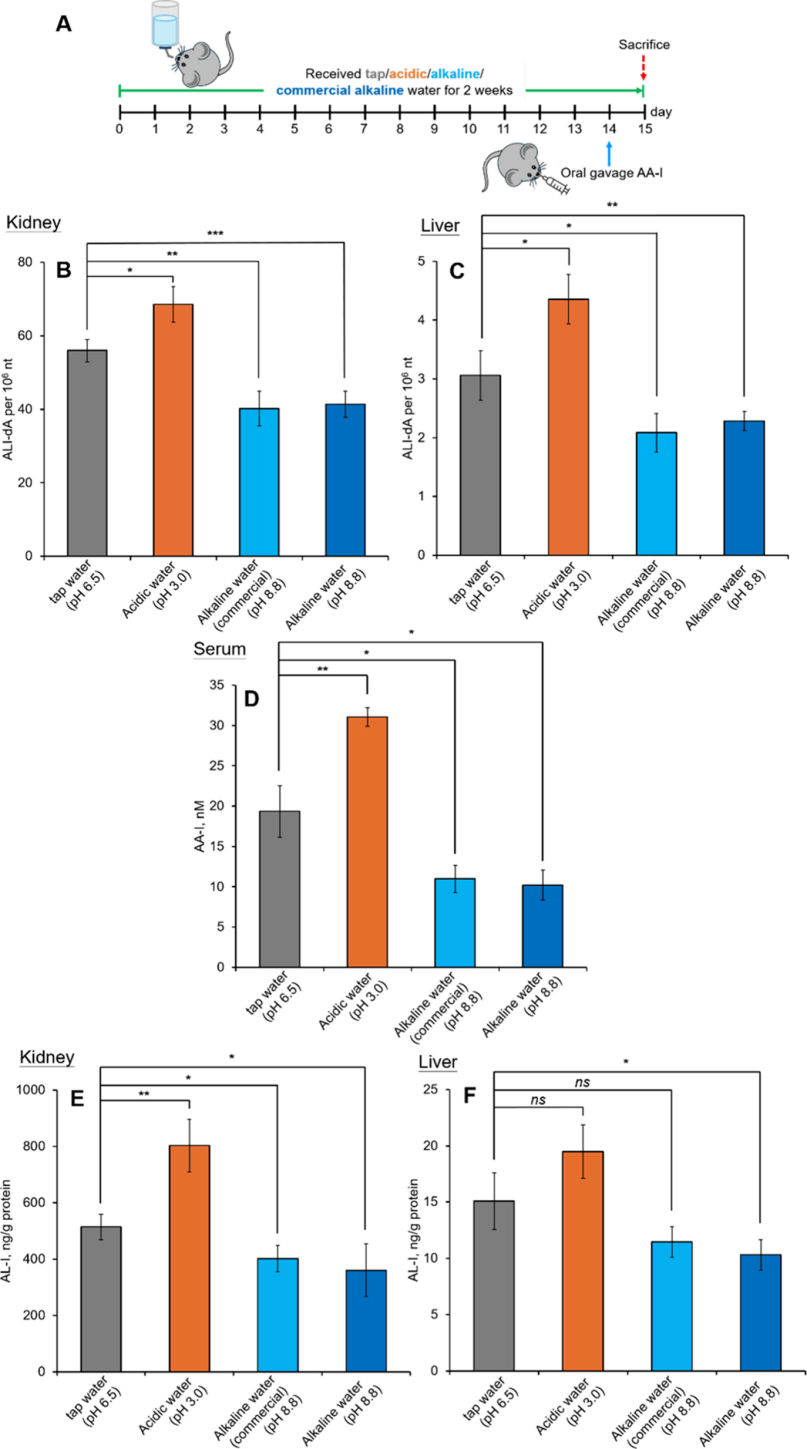
Effect of different pH levels of drinking water to the DNA adduct formation in aristolochic acid I exposed mice. (A) Experimental design for aristolochic acid I exposed mice fed the standard diet with drinking water of different pH levels. DNA adduct levels in the (B) kidney and (C) liver, (D) serum AA-I levels, and concentrations of aristolactam I in the (E) kidney and (F) liver of aristolochic acid I exposed mice fed the standard diet with drinking water of different pH levels. Data represent mean ± SD of five independent measurements and were compared with those in mice receiving tap water (B: 54.7 ± 3.0 adducts per 10^6^ nucleotides; C: 2.90 ± 0.42 adducts per 10^6^ nucleotides; D: 18.8 ± 3.2 nM; E: 510.2 ± 45.4 ng aristolactam I/g protein; and F: 14.2 ± 2.5 ng aristolactam I/g protein).

These observations were accompanied by changes in the intestinal pH (Figure S1). Measurements of intestinal fluid pH indicated that the drinking water influenced intestinal pH, which in turn shifted the equilibrium of AA between its neutral and deprotonated forms. The higher pH of the intestinal fluid resulting from alkaline drinking water favored the deprotonated ionic form of AA, enhancing its water solubility and urinary excretion.^13,27,55^ Consequently, less AA was absorbed into the hydrophobic bilayer of the intestinal wall via passive diffusion (Figure 5D), leading to a lower level of DNA adduct formation. The hypothesis that intestinal pH affected AA absorption was supported by our gut sac experiment using Tyrode’s solution buffered at different pH levels (Figure 3B).

Overall, this study revealed that an unbalanced diet and drinking acidified water increased the DNA adduct formation of AAs and thus the risk of developing BEN. These results highlights the importance of a balanced diet and the potential of using alkaline drinking water as a risk mitigation strategy for people living in affected areas.

## Supplementary Material



## Notes

Composition of the different mice diets used in the study; HPLC and mass spectrometers used for analyzing AA-I, AL-I, and ALI-dA; and pH of the intestinal fluid of mice drinking water of different pH levels (PDF)

## ABBREVIATIONS

AA: asaristolochic acids
AA-I: aristolochic acid I
AL-I: aristolactam I
ALI-dA: 7-(deoxyadenosin-N^6^-yl)-aristolactam I
ALI-dG: 7-(deoxyguanosin-N^2^-yl)-aristolactam I
LC–MS/MS: liquid chromatography–tandem mass spectrometry
