# Supplementary material for: Impact of Dietary Practices on DNA Adduct Formation by Aristolochic Acid I in Mice: Drinking Alkaline Water as a Risk Mitigation Strategy
Source: Chem Res Toxicol. 2025 Dec 8;39(1):95–103. doi: 10.1021/acs.chemrestox.5c00354 (PMC12820952; doi:10.1021/acs.chemrestox.5c00354)
Supplement: Supplementary file 1 [file tx5c00354_si_001.pdf]

Supporting Information

Impact of Dietary Practices on DNA Adduct Formation by Aristolochic Acid I in Mice: Drinking Alkaline Water as a Risk Mitigation Strategy

Hong-Ching Kwok [a, #], Jiayin Zhang [a, #], Nikola M. Pavlović [b], and Wan Chan [a,*]

[a] Department of Chemistry, The Hong Kong University of Science and Technology, Clear Water Bay, Kowloon, Hong Kong

[b] Medical Faculty, University of Niš, Bulevar Dr Zorana Đinđića 81, Niš 18000, Serbia

[#] the authors contributed equally to this work

[*] Corresponding authors: chanwan@ust.hk,

TABLE OF CONTENTS

Table S1. Composition of mice chews used in this study. (*Page S3*)

Table S2. LC gradient, MS source parameters, and MS compound parameters for LC-MS/MS analysis of (A) AA-DNA adducts, (B) AA-I and AL-I. (*Page S4 – S5*)

Figure S1. Effects of drinking water of different pH and the administration of $CaCO_3$ supplement on mice intestinal pH. (*Page S6*)

Table S1. Composition of mice chews used in this study. [a]

Ingredients (g/kg)	*Standard diet*	Fat, level 1	Fat, level 2	Protein, level 1	Protein, level 2	Salt, level 1	Salt, level 2	Sucrose, level 1	Sucrose, level 2
Casein	*197*	197	197	**297**	**397**	197	197	197	197
L-cystine	*3*	3	3	3	3	3	3	3	3
Corn starch	*168*	168	168	168	168	168	168	168	168
Maltodextrin	*132*	132	132	132	132	132	132	132	132
Sucrose	*0*	0	0	0	0	0	0	**100**	**200**
Cellulose	*352.5*	282.5	202.5	252.5	152.5	335.5	315.5	252.5	152.5
Soybean oil	*100*	100	100	100	100	100	100	100	100
Lard	*0*	**70**	**150**	0	0	0	0	0	0
Vitamin mix	*10*	10	10	10	10	10	10	10	10
Mineral mix	*35*	35	35	35	35	35	35	35	35
NaCl	*0*	0	0	0	0	**17**	**37**	0	0
Choline bitartrate	*2.5*	2.5	2.5	2.5	2.5	2.5	2.5	2.5	2.5
Total	*1000*	1000	1000	1000	1000	1000	1000	1000	1000

[a] The amount of cellulose was adjusted in different diets to maintain a constant mass concentration.

Table S2. LC gradient, MS source parameters, and MS compound parameters for LC-MS/MS analysis of (A) ALI-dA and ALI-dG adducts, (B) AA-I and AL-I.

(A) ALI-dA and ALI-dG adduct analysis

LC gradient

Time, min	Flow rate, mL/min	% A	% B
0.00	0.35	98.0	2.0
2.00	0.35	98.0	2.0
3.00	0.35	70.0	30.0
6.00	0.35	30.0	70.0
7.00	0.35	0.0	100.0
11.00	0.35	0.0	100.0
11.10	0.35	98.0	2.0
13.50	0.35	98.0	2.0

A: 0.1% acetic acid in water; B: acetonitrile

ESI source parameter

Capillary Voltage, kV	2
Source Temperature, ^{o}C	150
Desolvation Temperature, ^{o}C	500
Cone gas flow, L/h	300
Desolvation gas flow, L/h	1000

MS compound parameter

	Parent ion, *m/z*	Daughter ion, *m/z*	Cone voltage, V	Collision energy, eV
ALI-dA	543	427	40	20
	543	292	40	40
ALI-dG	559	443	20	18
	559	292	20	35
$^{15}N_5$-ALI-dA	548	432	40	20

(B) AA-I and AL-I analysis

LC gradient

Time, min	Flow rate, mL/min	% A	% B
0.00	0.40	90.0	10.0
1.00	0.40	90.0	10.0
8.00	0.40	30.0	70.0
8.10	0.40	0.0	100.0
10.50	0.40	0.0	100.0
10.60	0.40	90.0	10.0
12.00	0.40	90.0	10.0

A: 0.2% acetic acid and 0.01M ammonium acetate in water; B: acetonitrile

ESI source parameter

Curtain Gas, psi	25
Collision Gas	High
IonSpray Voltage, V	5500
Temperature, °C	500
Ion Source Gas 1, psi	30
Ion Source Gas 2, psi	60

MS compound parameter

	Parent ion, *m/z*	Daughter ion, *m/z*	[a]DP, V	[b]EP, V	[c]CE, V	[d]CXP, V
AA-I	359	324	50	10	20	15
	359	298	50	10	20	25
AL-I	294	279	100	12	50	15
	294	251	100	12	40	15
benz[*cd*]indol-2(1*H*)-one	170	115	110	5	50	11

[a]DP: Declustering Potential
[b]EP: Entrance Potential
[c]CE: Collision Energy
[d]CXP: Collision Cell Exit Potential

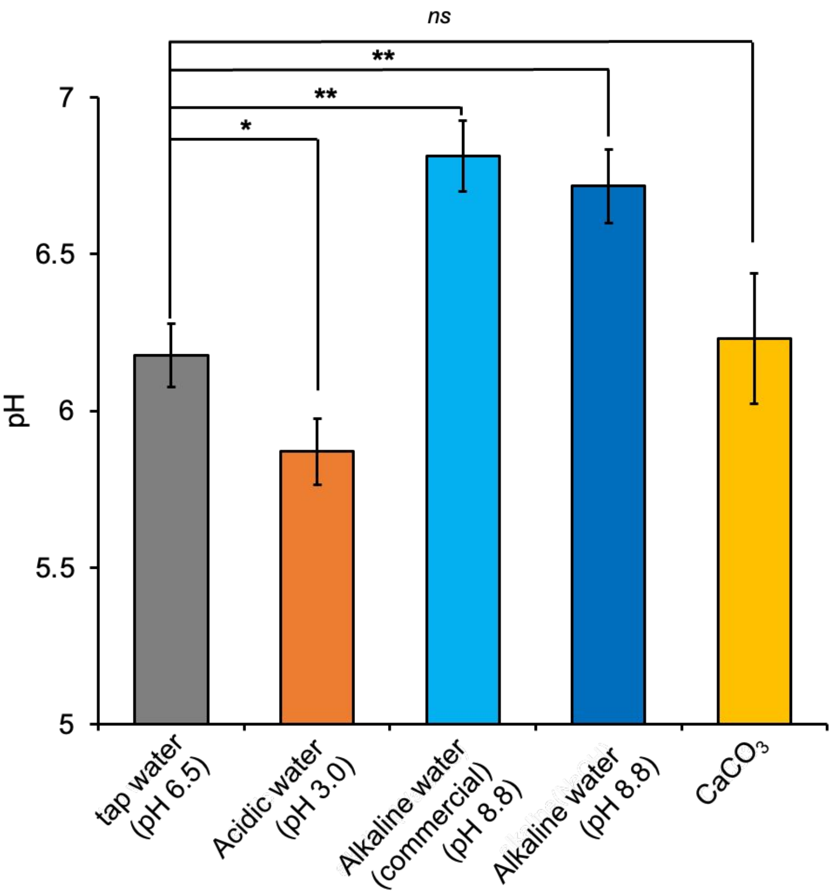

Figure S1. Effects of drinking water of different pH and the administration of $CaCO_3$ supplement on mice intestinal pH.
